# Biological Strategies to Enhance Healing of the Avascular Area of the Meniscus

**DOI:** 10.1155/2012/528359

**Published:** 2011-12-13

**Authors:** Umile Giuseppe Longo, Stefano Campi, Giovanni Romeo, Filippo Spiezia, Nicola Maffulli, Vincenzo Denaro

**Affiliations:** ^1^Department of Orthopaedic and Trauma Surgery, Campus Bio-Medico University, Via Alvaro del Portillo, 200, 00128 Rome, Italy; ^2^Centro Integrato di Ricerca (CIR), Università Campus Bio-Medico di Roma, Via Alvaro del Portillo, 21, 00128 Roma, Italy; ^3^Centre for Sports and Exercise Medicine, Barts and The London School of Medicine and Dentistry, Mile End Hospital, 275 Bancroft Road, London E1 4DG, UK

## Abstract

Meniscal injuries in the vascularized peripheral part of the meniscus have a better healing potential than tears in the central avascular zone because meniscal healing principally depends on its vascular supply. Several biological strategies have been proposed to enhance healing of the avascular area of the meniscus: abrasion therapy, fibrin clot, organ culture, cell therapy, and applications of growth factors. However, data are too heterogeneous to achieve definitive conclusions on the use of these techniques for routine management of meniscal lesions. Although most preclinical and clinical studies are very promising, they are still at an experimental stage. More prospective randomised controlled trials are needed to compare the different techniques for clinical results, applicability, and cost-effectiveness.

## 1. Introduction

The menisci of the knee are two semilunar fibrocartilaginous structures sitting between the joint surfaces of femoral condyles and the tibial plateau. Meniscal injuries are a common and important source of knee dysfunction. Repair should be considered depending on the type and location of the meniscal tear [1–3], as meniscal healing principally depends on the vascular supply of the zone that has been injured [4]. A rich network of arborizing vessels within the peripheral capsular and synovial attachments supplies vascularization to the menisci. This perimeniscal network provides radial branches to the meniscus. The outer third of the meniscus is vascularised, showing a good healing capacity. Given its abundant vascularization, this zone is also called “red-red zone.” The remaining two-thirds of the meniscus, respectively called “red-white zone” and “white-white zone,” have a scanty vascular supply and present a limited ability to heal spontaneously [4–7].

A meniscal lesion followed by disruption of the structure in the avascular zone impairs load distribution and initiates erosion of the adjacent articular surfaces, causing osteoarthritis (OA) [8–11]. The most common treatment for lesions of the avascular part of the meniscus is arthroscopic partial meniscectomy, which reduces symptoms but similarly predisposes patients to OA [12]. Studies have demonstrated that healing of the knee is inversely related to the amount of resected meniscal tissue [10, 11, 13, 14]. Meniscal repair techniques in the avascular zone are in continuous evolution.

This paper covers current knowledge on biological strategies for the stimulation of meniscal healing after repair.

## 2. Abrasion Therapy

Rasping of the damaged meniscus in the vascularized parameniscal synovium promotes an injury response and is one of the most simple and effective strategies to favour healing [3]. A small incision is performed to produce a vascular channel that redirects the blood flow from the vascular zone into the avascular one. Several studies showed a significant difference in healing between menisci treated with abrasion therapy and control groups [15, 16]. The most common techniques of abrasion therapy are rasping or trephination, in which radially oriented channels are performed to encourage vascular and cellular migration from the peripheral vascular portion to the tear site [17–19]. Rasping increases the production of interleukin-I-alpha (IL-1-alpha), transforming-growth-factor-beta 1 (TGF-beta1), platelet-derived growth factor (PDGF), and proliferating cell nuclear antigen (PCNA). This protein network improves vascular induction and meniscal healing [20]. Nevertheless, trephination and rasping procedures may damage the normal meniscal structure by an additional full thickness-transverse tear, resulting in poor meniscal function.

## 3. Fibrin Clot

Fibrin is a fibrous protein produced in response to bleeding that plays an important role in blood clotting. Fibrin clots may be used topically or by injection as an haemostatic agent, binding to several adhesive proteins of different cells [21–33]. The fibrin clot technique acts as a chemotactic and mitogenic stimulus for reparative cells because of the presence of several growth factors [34–37]. The fibrin clot attaches to the exposed collagen caused by the tear and induces proliferation of fibrous connective tissue [38–44]. This stimulates the development of fibrocartilaginous tissue. The fibrin clot technique can be used in combination with abrasion therapy or with meniscal sutures [45–57]. Two studies in animal models showed that organized fibrous connective tissue developed into cartilaginous tissue after a period of 12–24 weeks [58, 59]. A potential disadvantage of the fibrin clot technique is the difficulty of keeping fibrin clots on the tear without immobilizing the operated leg [60].

## 4. Organ Culture

Organ culture is a useful model to assess the intrinsic healing potential of the meniscus excluding the influence of microvasculature and the synovium [61–63]. The effects of cultured meniscal explants in a rabbit model have been reported [63]. After gross evaluation, each meniscal explant underwent histological evaluation to study the relationship between the graft and recipient tissue. Application of this technique has demonstrated that meniscal tissue presents an intrinsic healing ability, which is greater in the peripheral zone of the meniscus than in the inner zone [63]. Regional differences in healing potential and extrinsic factors, such as blood supply, could explain good meniscal healing in the peripheral zone.

## 5. Cell Therapy

Human menisci are populated by different cell types, responding differently to various stimuli released from the matrix [64, 65]. Different cells have already been used in studies on meniscal healing: mesenchymal stem cells (MSCs) deriving from synovial or bone marrow, chondrocytes, and fibrochondrocytes. MSCs are pluripotent cells able to differentiate into specific therapeutic cell types (developmental plasticity) [66–68]. The effects of bioactive molecules, which are secreted by MSCs, determine a regenerative microenvironment that promotes healing of meniscal lesions [69, 70]. The combination of suturing and MSC treatment, combined or not with fibrin glue, seems to be the most effective treatment [71].

Zellner et al. [70] reported the efficacy of mesenchymal stem cells in the repair of meniscal defects in the avascular zone. Nonprecultured mesenchymal stem cells in hyaluronan-collagen composite matrices stimulated the development of completely integrated meniscus-like repair tissue in defects produced in the avascular zone of rabbit menisci [70].

Further studies confirm the production of abundant extracellular matrix around the cells, restoring a meniscal-like tissue in the avascular zone [70, 72–75]. These results are supported by early studies which demonstrated the efficacy of the association between growth factors and mesenchymal stem cells within scaffold implants to increase proteoglycans and/or collagen synthesis [70, 76]. Articular autologous and allogenic chondrocytes have also been used to induce repair in the avascular part of the meniscus [77, 78]. Peretti et al. described a porcine chondrocyte model where implantation of such cells was performed in the avascular part of the meniscus using an allogenic scaffold seeded with autologous chondrocytes. These chondrocytes were effective in promoting healing meniscal tears [77]. Fibrochondrocytes showed potential for initiating a reparative response in meniscal defects through the production of new extracellular matrix (ECM) [79, 80]. When seeded into a porous collagen scaffold, fibrochondrocytes harvested from the inner avascular part of the meniscus produce more glycosaminoglycans (GAGs) than fibrochondrocytes from a peripheral fibrous location [81, 82]. Although these findings are encouraging, the application of autologous fibrochondrocytes in meniscal tissue engineering is limited by the difficulty in harvesting a sufficient number of cells [83–93].

## 6. Growth Factors

Growth factors act as signalling molecules on target cells to stimulate the regeneration of damaged tissue [6]. Furthermore, they can induce the synthesis and inhibit degradation of ECM by a mechanism of downregulation of proteases [94]. Several studies in vitro and in vivo evaluated the effects of treatment with specific growth factors. Two categories of growth factors in consideration of their biochemical attributes are generally considered: anabolic and catabolic growth factors.

### 6.1. Anabolic Growth Factors

#### 6.1.1. Fibroblast Growth Factor (FGF)

basic FGF was used to stimulate type II collagen and aggrecan mRNA production in cellular and tissue development [95, 96]. In an ovine experimental model, meniscal fibrochondrocytes responded to bFGF by proliferating and producing new extracellular matrix [79]. Another FGF type, FGF-2, stimulates proliferation of the joint chondrocytes, mesenchymal stem cells, osteoblasts, and adipocytes. Furthermore, it maintains the ability of any cell types to differentiate [97, 98]. Moreover, a hyperexpression of FGF-2 and alpha-smooth muscle actin (alpha-SMA) through recombinant adeno-associated virus (rAAV) enhanced cell proliferation and increased survival rate compared with control groups. However, FGF did not significantly increase the synthesis of major extracellular matrix components or DNA contents [99].

#### 6.1.2. Transforming-Growth-Factor-Beta (TGF-Beta)

TGF-beta seems to have several regulatory activities, stimulating collagen and proteoglycan production to increase the attachment of the cells in repaired meniscal tissue. Nevertheless, it has no effect on cell proliferation [6, 100–103].

#### 6.1.3. Bone Morphogenetic Proteins (BMPs)

BMPs are a group of growth factors belonging to the TGF-*β* superfamily playing an important role during embryogenesis and tissue repair in relation to their osteoinductive properties [104, 105]. BMP-2 acts as a stimulus in the differentiation of mesenchymal cells. It also presents a migratory effect in endothelial cells or smooth muscle cells, but rarely in chondrocytes [106]. BMP-7 regulates matrix homeostasis and inhibits the processes of degradation. BMP-7 acts with different chondrogenic agents and is more effective than BMP-2 in chondrogenic differentiation of MSCs in promoting meniscal healing [107].

#### 6.1.4. Insulin-Like-Growth-Factor-I (IGF-I)

This is considered the main anabolic growth factor for articular cartilage [108, 109]. Unlike TGF-beta I, IGF-I increases cell proliferation significantly but has no effect on the attachment [95]. Therefore, a mixture of growth factors in association with IGF-I could induce an extensive cellular response to mediate avascular meniscal healing [100].

#### 6.1.5. Vascular Endothelial Growth Factor (VEGF)

The induction of angiogenesis is important to stimulate healing of meniscal tears [110–122]. Vascular endothelial growth factor (VEGF) may promote better healing, stimulating angiogenesis to improve the healing capacities of meniscus tissue. In adults, VEGF expression is downregulated by endostatin, mostly in the avascular zone [123]. However, the local application of VEGF did not show an improvement of meniscal healing [124].

#### 6.1.6. Platelet-Derived Growth Factor-AB (PDGF-AB)

PDGF-AB plays an important role in the angiogenesis and cell development [125, 126]. The application of PDGF-AB in the peripheral part of the menisci showed a better healing response than the application in the central part [127]. However, this anabolic growth factor increased both cell proliferation and ECM formation in all zones of the meniscus, including the avascular zone [128].

### 6.2. Catabolic Growth Factors

#### 6.2.1. Endostatin

Endostatin is an antiangiogenic factor expressed by fibrochondrocytes in the avascular zone of menisci. Endostatin concentrations were higher when fibrochondrocytes were in coculture with MSCs, suggesting that meniscal cell growth is inhibited by the proliferation of MSCs [7].

#### 6.2.2. Interleukin-I (IL-I)

This is a proinflammatory cytokine that stimulates the development of a local inflammatory reaction. Meniscal explants treated with IL-I have failed to show any signs of regeneration [129]. These findings suggest that relevant expression of IL-I in association with higher levels of tumor-necrosis-factor-alpha (TNF-alpha) inhibit meniscal repair [130].

## 7. Platelet Rich Plasma

Platelet-rich plasma (PRP) is an autologous substance rich in platelets that releases growth factors from both alpha and dense granules [131–150]. These growth factors have been associated with the initiation of a healing cascade leading to cellular chemotaxis, angiogenesis, collagen matrix synthesis, and cell proliferation [151]. Ishida et al. reported the effects of PRP on meniscal tissue regeneration, both in vitro and in vivo, in a rabbit model. In the in vitro study, monolayer meniscal cell cultures were prepared and proliferative behaviour, extracellular matrix (ECM) synthesis, and fibrocartilage-related messenger ribonucleic acid (mRNA) expressions were assessed in the presence of PRP. PRP stimulated DNA synthesis, ECM synthesis, and mRNA expression of biglycan and decorin [152–161]. In the *in vivo * study, full-thickness defects were produced in the avascular region of rabbit meniscus. Gelatin hydrogel (GH) was used to deliver PRP into the defects. At histology 12 weeks after surgery, significantly better meniscal repair was evident in animals that received PRP with GH than in the control groups [162].

In contrast, Zellner et al. evaluated several cell and biomaterial-based treatment options for repair of defects in the avascular zone of rabbit menisci by producing circular meniscal punch defects in the avascular zone of rabbit menisci. The defects were left empty or filled with hyaluronan-collagen composite matrices without cells loaded with platelet-rich plasma, autologous bone marrow, or autologous mesenchymal stem cells. Neither bone marrow nor platelet-rich plasma loaded in matrices induced improvement in meniscal healing [70].

## 8. Discussion

In the last few decades, many studies on meniscal healing have focused on methods to enhance the healing capacities of the meniscus after repair [163–178]. Abrasion of the torn meniscus and synovial tissue or the establishment of vascular channels to redirect blood flow into the avascular zone seems to be the preferred treatment [3, 15, 16]. However, the healing potential depends on the type and location of the tear and its distance from the peripheral vascularised zone. The use of a fibrin clot can also be an effective technique to support a reparative response in the avascular zone of the meniscus [34, 35, 37]. Findings demonstrated that the rasping technique is more effective than fibrin clot application to improve meniscal healing [179]. Kobayashi et al. reported healing rates in the peripheral zone of the menisci in an on-organ culture model. Regional differences in healing potential and extrinsic factors, such as a blood supply, could explain the good meniscal healing potential in the peripheral zone [63].

Cell-based therapy for meniscal tears has significantly contributed to an increasing number of patients treated with repair techniques rather than meniscectomy. Different cell types have already been used in studies on meniscus healing: MSCs, articular chondrocytes and autologous fibrochondrocytes [70, 77, 81, 82]. Progenitor cells such as mesenchymal stem cells present the advantage of being easily expandable without losing their differentiation potential into a variety of mesenchymal tissues including bone, tendon, cartilage, muscle, ligament, fat, and marrow stroma [64, 66, 68]. The application of MSCs and their stimulation with growth factors in combination with a mechanically loadable scaffold have been proposed as the focus of future studies [135, 148].

Several studies reported the efficacy of mesenchymal stem cells in the repair of meniscal defects in the avascular zone, with production of abundant extracellular matrix around the cells and restoration of a meniscal-like tissue [70, 72–75]. Early studies demonstrated the efficacy of the association between growth factors and mesenchymal stem cells within scaffold implants to increase proteoglycan and/or collagen synthesis [180–196]. Therefore, the healing response of mesenchymal stem cells seems to produce additional repair qualities besides the delivery of growth factors [70, 76].

Many studies have shown the importance of growth factors in the treatment of meniscal tears of the avascular portion, but there is a very complex interplay among a variety of factors that influences healing processes. Growth factors that promote cell differentiation and chondrocytic proliferation include both anabolic growth factors (TGF-beta I, BMPs, IGF-I, FGF, VEGF, and PDGF-AB) and catabolic growth factors (endostatin, IL-1, and TNF-alpha). Anabolic growth factors could be of additional value in improving the healing of meniscal lesions [6, 79, 95–109, 123–128]. However, the application of growth factors remains very limited in clinical settings [6, 95]. Future research should focus on the use of tissue-engineered constructs in association with different growth factors [197–203]. A preparation rich in growth factors could produce better results than the use of isolated growth factors. Only a few studies to date have evaluated the effectiveness of a preparation of platelet-rich plasma (PRP), but there is some evidence that PRP can improve healing of the menisci [127, 128]. The release of growth factors from platelets has been associated with the initiation of a healing cascade leading to cellular chemotaxis, angiogenesis, collagen matrix synthesis, and cell proliferation [151, 162]. In contrast, a study in an animal model reported that application of PRP did not produce improvements in meniscal healing [70].

## 9. Conclusion

Patients with meniscal tears report pain and functional limitation of the knee joint. Partial meniscectomy is the most common treatment option, but it represents a predisposing factor for osteoarthritis [12]. To date only limited scientifically proven management modalities are available. A better understanding of meniscal healing mechanisms will allow specific treatment strategies to be developed. Although most preclinical and clinical studies are very promising, they are still at an experimental stage. Further prospective trials are necessary to compare the different techniques for efficacy, applicability, and cost-effectiveness in the management of lesions of the avascular region of the meniscus.

## References

[1] Rath E, Richmond JC (2000). The menisci: basic science and advances in treatment. *British Journal of Sports Medicine*.

[2] Messner K, Gao J (1998). The menisci of the knee joint. Anatomical and functional characteristics, and a rationale for clinical treatment. *Journal of Anatomy*.

[3] Arnoczky SP, Warren RF (1983). The microvasculature of the meniscus and its response to injury. An experimental study in the dog. *American Journal of Sports Medicine*.

[4] Arnoczky SP, Warren RF (1982). microvasculature of the human meniscus. *American Journal of Sports Medicine*.

[5] Ghadially FN, Lalonde JMA, Wedge JH (1983). Ultrastructure of normal and torn menisci of the human knee joint. *Journal of Anatomy*.

[6] Forriol F (2009). Growth factors in cartilage and meniscus repair. *Injury*.

[7] Hoberg M, Schmidt EL, Tuerk M, Stark V, Aicher WK, Rudert M (2009). Induction of endostatin expression in meniscal fibrochondrocytes by co-culture with endothelial cells. *Archives of Orthopaedic and Trauma Surgery*.

[8] Krause WR, Pope MH, Johnson RJ, Wilder DG (1976). Mechanical changes in the knee after meniscectomy. *Journal of Bone and Joint Surgery—Series A*.

[9] Levy IM, Torzilli PA, Gould JD, Warren RF (1989). The effect of lateral meniscectomy on motion of the knee. *Journal of Bone and Joint Surgery—Series A*.

[10] Roos H, Laurén M, Adalberth T, Roos EM, Jonsson K, Lohmander LS (1998). Knee osteoarthritis after meniscectomy: prevalence of radiographic changes after twenty-one years, compared with matched controls. *Arthritis and Rheumatism*.

[11] Roos H, Adalberth T, Dahlberg L, Lohmander LS (1995). Osteoarthritis of the knee after injury to the anterior cruciate ligament or meniscus: the influence of time and age. *Osteoarthritis and Cartilage*.

[12] Forriol F, Longo UG, Hernández-Vaquero D (2010). The effects of previous meniscus and anterior cruciate ligament injuries in patients with total knee arthroplasty. *Ortopedia Traumatologia Rehabilitacja*.

[13] McDermott ID, Amis AA (2006). The consequences of meniscectomy. *Journal of Bone and Joint Surgery—Series B*.

[14] Englund M, Guermazi A, Lohmander LS (2009). The meniscus in knee osteoarthritis. *Rheumatic Disease Clinics of North America*.

[15] Gershuni DH, Skyhar MJ, Danzig LA, Camp J, Hargens AR, Akeson WH (1989). Experimental models to promote healing of tears in the avascular segment of canine knee menisci. *Journal of Bone and Joint Surgery—Series A*.

[16] Okuda K, Ochi M, Shu N, Uchio Y (1999). Meniscal rasping for repair of meniscal tear in the avascular zone. *Arthroscopy*.

[17] Zhang Z, Tu K, Xu Y, Zhang W, Liu Z, Ou S (1988). Treatment of longitudinal injuries in avascular area of meniscus in dogs by trephination. *Arthroscopy*.

[18] Zhang Z, Arnold JA, Willlams T, McCann B, Cannon WD (1995). Repairs by trephination and suturing of longitudinal injuries in the avascular area of the meniscus in goats. *American Journal of Sports Medicine*.

[19] Cook JL, Fox DB (2007). A novel bioabsorbable conduit augments healing of avascular meniscal tears in a dog model. *American Journal of Sports Medicine*.

[20] Ochi M, Uchio Y, Okuda K, Shu N, Yamaguchi H, Sakai Y (2001). Expression of cytokines after meniscal rasping to promote meniscal healing. *Arthroscopy*.

[21] Ames PRJ, Longo UG, Denaro V, Maffulli N (2008). Achilles tendon problems: not just an orthopaedic issue. *Disability and Rehabilitation*.

[22] Amlang MH, Maffuli N, Longo G, Stübig T, Imrecke J, Hüfner T (2010). Surgical treatment of Achilles tendon rupture. *Unfallchirurg*.

[23] Becher C, Driessen A, Hess T, Longo UG, Maffulli N, Thermann H (2010). Microfracture for chondral defects of the talus: maintenance of early results at midterm follow-up. *Knee Surgery, Sports Traumatology, Arthroscopy*.

[24] Capuano L, Hardy P, Longo UG, Denaro V, Maffulli N (2008). No difference in clinical results between femoral transfixation and bio-interference screw fixation in hamstring tendon ACL reconstruction. A preliminary study. *Knee*.

[25] Castricini R, Longo UG, De Benedetto M (2011). Platelet-rich plasma augmentation for arthroscopic rotator cuff repair: a randomized controlled trial. *American Journal of Sports Medicine*.

[26] Chimutengwende-Gordon M, Khan WS, Sidhu J, Longo UG, Maruthainar N (2010). Advanced trauma life support radiographic trauma series: part 2—the chest radiograph. *Journal of perioperative practice*.

[27] De Mozzi P, Longo UG, Galanti G, Maffulli N (2008). Bicuspid aortic valve: a literature review and its impact on sport activity. *British Medical Bulletin*.

[28] Denaro V, Di Martino A, Longo UG (2008). Effectiveness of a mucolythic agent as a local adjuvant in revision lumbar spine surgery. *European Spine Journal*.

[29] Denaro L, Longo UG, Papalia R, Di Martino A, Maffulli N, Denaro V (2008). Eosinophilic granuloma of the pediatric cervical spine. *Spine*.

[30] Denaro L, Longo UG, Denaro V (2009). Vertebroplasty and kyphoplasty: reasons for concern?. *Orthopedic Clinics of North America*.

[31] Denaro V, Ruzzini L, Longo UG (2010). Effect of dihydrotestosterone on cultured human tenocytes from intact supraspinatus tendon. *Knee Surgery, Sports Traumatology, Arthroscopy*.

[32] Denaro V, Longo UG, Denaro L (2010). Vertebroplasty versus conservative treatment for vertebral fractures. *The Lancet*.

[33] Denaro V, Ruzzini L, Barnaba SA (2011). Effect of pulsed electromagnetic fields on human tenocyte cultures from supraspinatus and quadriceps tendons. *American Journal of Physical Medicine and Rehabili*.

[34] Arnoczky SP, Warren RF, Spivak JM (1988). Meniscal repair using an exogenous fibrin clot. An experimental study in dogs. *Journal of Bone and Joint Surgery—Series A*.

[35] Henning CE, Lynch MA, Yearout KM, Vequist SW, Stallbaumer RJ, Decker KA (1990). Arthroscopic meniscal repair using an exogenous fibrin clot. *Clinical Orthopaedics and Related Research*.

[36] Ishimura M, Tamai S, Fujisawa Y (1991). Arthroscopic meniscal repair with fibrin glue. *Arthroscopy*.

[37] van Trommel MF, Simonian PT, Potter HG, Wickiewicz TL (1998). Arthroscopic meniscal repair with fibrin clot of complete radial tears of the lateral meniscus in the avascular zone. *Arthroscopy*.

[38] Forriol F, Longo UG, Pueyo J, Maffulli N, Denaro V (2009). Computed tomography-based study of age- and sex-related variation in morphology of the femur. *Ortopedia Traumatologia Rehabilitacja*.

[39] Forriol F, Longo UG, Concejo C, Ripalda P, Maffulli N, Denaro V (2009). Platelet-rich plasma, rhOP-1 (rhBMP-7) and frozen rib allograft for the reconstruction of bony mandibular defects in sheep. A pilot experimental study. *Injury*.

[40] Forriol F, Denaro L, Longo UG, Taira H, Maffulli N, Denaro V (2010). Bone lengthening osteogenesis, a combination of intramembranous and endochondral ossification: an experimental study in sheep. *Strategies in Trauma and Limb Reconstruction*.

[41] Forriol F, Longo UG, Alvarez E (2011). Scanty integration of osteochondral allografts cryopreserved at low temperatures with dimethyl sulfoxide. *Knee Surgery, Sports Traumatology, Arthroscopy*.

[42] Franceschi F, Marinozzi A, Papalia R, Longo UG, Gualdi G, Denaro E (2006). Intra- and juxta-articular osteoid osteoma: a diagnostic challenge. *Archives of Orthopaedic and Trauma Surgery*.

[43] Franceschi F, Longo UG, Ruzzini L, Rizzello G, Denaro V (2007). Arthroscopic management of calcific tendinitis of the subscapularis tendon. *Knee Surgery, Sports Traumatology, Arthroscopy*.

[44] Franceschi F, Longo UG, Ruzzini L (2007). Dislocation of an enlarged fabella as uncommon cause of knee pain. A case report. *Knee*.

[45] Franceschi F, Ruzzini L, Longo UG (2007). Equivalent clinical results of arthroscopic single-row and double-row suture anchor repair for rotator cuff tears: a randomized controlled trial. *American Journal of Sports Medicine*.

[46] Franceschi F, Longo UG, Ruzzini L, Denaro V (2007). Isolated tuberculosis of the patellar tendon. *Journal of Bone and Joint Surgery—Series B*.

[47] Franceschi F, Longo GU, Ruzzini L, Rizzello G, Maffulli N, Denaro V (2007). The Roman Bridge: a “double pulley-suture bridges” technique for rotator cuff repair. *BMC Musculoskeletal Disorders*.

[48] Franceschi F, Longo UG, Ruzzini L, Papalia R, Rizzello G, Denaro V (2007). To detach the long head of the biceps tendon after tenodesis or not: outcome analysis at the 4-year follow-up of two different techniques. *International Orthopaedics*.

[49] Franceschi F, Longo UG, Ruzzini L, Rizzello G, Maffulli N, Denaro V (2008). Arthroscopic salvage of failed arthroscopic Bankart repair: a prospective study with a minimum follow-up of 4 years. *American Journal of Sports Medicine*.

[50] Franceschi F, Longo UG, Ruzzini L (2008). Circulating substance P levels and shoulder joint contracture after arthroscopic repair of the rotator cuff. *British Journal of Sports Medicine*.

[51] Franceschi F, Longo UG, Ruzzini L (2008). En-bloc retrograde resection of an osteoid osteoma of the patella using computed tomography under arthroscopic control. *The Journal of Knee Surgery*.

[52] Franceschi F, Giuseppe Longo U, Ruzzini L, Rizzello G, Maffulli N, Denaro V (2008). No advantages in repairing a type II superior labrum anterior and posterior (SLAP) lesion when associated with rotator cuff repair in patients over age 50: a randomized controlled trial. *American Journal of Sports Medicine*.

[53] Franceschi F, Longo UG, Ruzzini L, Papalia R, Maffulli N, Denaro V (2008). Quadriceps tendon-patellar bone autograft for anterior cruciate ligament reconstruction: a technical note. *Bulletin of the NYU Hospital for Joint Diseases*.

[54] Franceschi F, Longo UG, Ruzzini L, Marinozzi A, Maffulli N, Denaro V (2008). Simultaneous arthroscopic implantation of autologous chondrocytes and high tibial osteotomy for tibial chondral defects in the varus knee. *Knee*.

[55] Franceschi F, Longo UG, Ruzzini L, Rizzello G, Maffulli N, Denaro V (2008). Soft tissue tenodesis of the long head of the biceps tendon associated to the Roman Bridge repair. *BMC Musculoskeletal Disorders*.

[56] Garau G, Rittweger J, Mallarias P, Longo UG, Maffulli N (2008). Traumatic patellar tendinopathy. *Disability and Rehabilitation*.

[57] Giombini A, Dragoni S, Averna T, Ripani M, Longo UG, Maffulli N (2009). Osteoid osteoma mimicking overuse syndromes in athletes. *Journal of Sports Medicine and Physical Fitness*.

[58] Hashimoto J, Kurosaka M, Yoshiya S, Hirohata K (1992). Meniscal repair using fibrin sealant and endothelial cell growth factor. An experimental study in dogs. *American Journal of Sports Medicine*.

[59] Port J, Jackson DW, Lee TQ, Simon TM (1996). Meniscal repair supplemented with exogenous fibrin clot and autogenous cultured marrow cells in the goat model. *American Journal of Sports Medicine*.

[60] Bray RC, Smith JA, Eng MK, Leonard CA, Sutherland CA, Salo PT (2001). Vascular response of the meniscus to injury: effects of immobilization. *Journal of Orthopaedic Research*.

[61] Ochi M, Mochizuki Y, Deie M, Ikuta Y (1996). Augmented meniscal healing with free synovial autografts: an organ culture model. *Archives of Orthopaedic and Trauma Surgery*.

[62] Mitani I, Sumita S, Takahashi N, Ochiai H, Ishii M (1996). 123I-MIBG myocardial imaging in hypertensive patients: abnormality progresses with left ventricular hypertrophy. *Annals of Nuclear Medicine*.

[63] Kobayashi K, Fujimoto E, Deie M, Sumen Y, Ikuta Y, Ochi M (2004). Regional differences in the healing potential of the meniscus—an organ culture model to eliminate the influence of microvasculature and the synovium. *Knee*.

[64] Verdonk PCM, Forsyth RG, Wang J (2005). Characterisation of human knee meniscus cell phenotype. *Osteoarthritis and Cartilage*.

[65] Stärke C, Kopf S, Petersen W, Becker R (2009). Meniscal repair. *Arthroscopy*.

[66] Ohishi M, Schipani E (2010). Bone marrow mesenchymal stem cells. *Journal of Cellular Biochemistry*.

[67] Nöth U, Osyczka AM, Tuli R, Hickok NJ, Danielson KG, Tuan RS (2002). Multilineage mesenchymal differentiation potential of human trabecular bone-derived cells. *Journal of Orthopaedic Research*.

[68] Oreffo ROC, Cooper C, Mason C, Clements M (2005). Mesenchymal stem cells lineage, plasticity, and skeletal therapeutic potential. *Stem Cell Reviews*.

[69] Cook JL (2005). The current status of treatment for large meniscal defects. *Clinical Orthopaedics and Related Research*.

[70] Zellner J, Mueller M, Berner A (2010). Role of mesenchymal stem cells in tissue engineering of meniscus. *Journal of Biomedical Materials Research—Part A*.

[71] Abdel-Hamid M, Hussein MR, Ahmad AF, Elgezawi EM (2005). Enhancement of the repair of meniscal wounds in the red-white zone (middle third) by the injection of bone marrow cells in canine animal model. *International Journal of Experimental Pathology*.

[72] Izuta Y, Ochi M, Adachi N, Deie M, Yamasaki T, Shinomiya R (2005). Meniscal repair using bone marrow-derived mesenchymal stem cells: experimental study using green fluorescent protein transgenic rats. *Knee*.

[73] Stone KR, Rodkey WG, Webber R, McKinney L, Steadman JR (1992). Meniscal regeneration with copolymeric collagen scaffolds. In vitro and in vivo studies evaluated clinically, histologically, and biochemically. *American Journal of Sports Medicine*.

[74] Steinert AF, Palmer GD, Capito R (2007). Genetically enhanced engineering of meniscus tissue using ex vivo delivery of transforming growth factor-*β*1 complementary deoxyribonucleic acid. *Tissue Engineering*.

[75] Dutton AQ, Choong PF, Goh JCH, Lee EH, Hui JHP (2010). Enhancement of meniscal repair in the avascular zone using mesenchymal stem cells in a porcine model. *Journal of Bone and Joint Surgery—Series B*.

[76] Pabbruwe MB, Kafienah W, Tarlton JF, Mistry S, Fox DJ, Hollander AP (2010). Repair of meniscal cartilage white zone tears using a stem cell/collagen-scaffold implant. *Biomaterials*.

[77] Peretti GM, Gill TJ, Xu JW, Randolph MA, Morse KR, Zaleske DJ (2004). Cell-based therapy for meniscal repair: a large animal study. *American Journal of Sports Medicine*.

[78] Weinand C, Peretti GM, Adams SB, Randolph MA, Savvidis E, Gill TJ (2006). Healing potential of transplanted allogeneic chondrocytes of three different sources in lesions of the avascular zone of the meniscus: a pilot study. *Archives of Orthopaedic and Trauma Surgery*.

[79] Tumia NS, Johnstone AJ (2004). Promoting the proliferative and synthetic activity of knee meniscal fibrochondrocytes using basic fibroblast growth factor in vitro. *American Journal of Sports Medicine*.

[80] Vander Schilden JL, York JL, Webber RJ (1991). Age-dependent fibrin clot invasion by human meniscal fibrochondrocytes. A preliminary report. *Orthopaedic Review*.

[81] Nakata K, Shino K, Hamada M (2001). Human meniscus cell: characterization of the primary culture and use for tissue engineering. *Clinical Orthopaedics and Related Research*.

[82] Tanaka T, Fujii K, Kumagae Y (1999). Comparison of biochemical characteristics of cultured fibrochondrocytes isolated from the inner and outer regions of human meniscus. *Knee Surgery, Sports Traumatology, Arthroscopy*.

[83] Khan WS, Longo UG (2011). ACI and MACI procedures for cartilage repair utilise mesenchymal stem cells rather than chondrocytes. *Medical Hypotheses*.

[84] Khanna A, Gougoulias N, Longo UG, Maffulli N (2009). Minimally invasive total knee arthroplasty: a systematic review. *Orthopedic Clinics of North America*.

[85] Khanna A, Friel M, Gougoulias N, Longo UG, Maffulli N (2009). Prevention of adhesions in surgery of the flexor tendons of the hand: what is the evidence?. *British Medical Bulletin*.

[86] Knobloch K, Schreibmueller L, Longo UG, Vogt PM (2008). Eccentric exercises for the management of tendinopathy of the main body of the Achilles tendon with or without an AirHeel*™* Brace. A randomized controlled trial. B: effects of compliance. *Disability and Rehabilitation*.

[87] Knobloch K, Schreibmueller L, Longo UG, Vogt PM (2008). Eccentric exercises for the management of tendinopathy of the main body of the Achilles tendon with or without the AirHeel*™* Brace. A randomized controlled trial. A: effects on pain and microcirculation. *Disability and Rehabilitation*.

[88] Lippi G, Longo UG, Maffulli N (2010). Genetics and sports. *British Medical Bulletin*.

[89] Longo G, Ripalda P, Denaro V, Forriol F (2006). Morphologic comparison of cervical, thoracic, lumbar intervertebral discs of cynomolgus monkey (Macaca fascicularis). *European Spine Journal*.

[90] Longo UG, Franceschi F, Ruzzini L (2007). Light microscopic histology of supraspinatus tendon ruptures. *Knee Surgery, Sports Traumatology, Arthroscopy*.

[91] Longo UG, King JB, Denaro V, Maffulli N (2008). Double-bundle arthroscopic reconstruction of the anterior cruciate ligament: does the evidence add up?. *Journal of Bone and Joint Surgery—Series B*.

[92] Longo UG, Franceschi F, Ruzzini L (2008). Histopathology of the supraspinatus tendon in rotator cuff tears. *American Journal of Sports Medicine*.

[93] Longo UG, Ramamurthy C, Denaro V, Maffulli N (2008). Minimally invasive stripping for chronic Achilles tendinopathy. *Disability and Rehabilitation*.

[94] Arnoczky SP (1999). Building a meniscus: biologic considerations. *Clinical Orthopaedics and Related Research*.

[95] Fox DB, Warnock JJ, Stoker AM, Luther JK, Cockrell M (2010). Effects of growth factors on equine synovial fibroblasts seeded on synthetic scaffolds for avascular meniscal tissue engineering. *Research in Veterinary Science*.

[96] Vincent T, Hermansson M, Bolton M, Wait R, Saklatvala J (2002). Basic FGF mediates an immediate response of articular cartilage to mechanical injury. *Proceedings of the National Academy of Sciences of the United States of America*.

[97] Narita A, Takahara M, Ogino T, Fukushima S, Kimura Y, Tabata Y (2009). Effect of gelatin hydrogel incorporating fibroblast growth factor 2 on human meniscal cells in an organ culture model. *Knee*.

[98] Martin I, Muraglia A, Campanile G, Cancedda R, Quarto R (1997). Fibroblast growth factor-2 supports ex vivo expansion and maintenance of osteogenic precursors from human bone marrow. *Endocrinology*.

[99] Cucchiarini M, Schetting S, Terwilliger EF, Kohn D, Madry H (2009). rAAV-mediated overexpression of FGF-2 promotes cell proliferation, survival, and *α*-SMA expression in human meniscal lesions. *Gene Therapy*.

[100] Izal I, Ripalda P, Acosta CA, Forriol F (2008). In vitro healing of avascular meniscal injuries with fresh and frozen plugs treated with TGF-beta1 and IGF-1 in sheep. *International Journal of Clinical and Experimental Pathology*.

[101] Collier S, Ghosh P (1995). Effects of transforming growth factor beta on proteoglycan synthesis by cell and explant cultures derived from the knee joint meniscus. *Osteoarthritis and Cartilage*.

[102] Pangborn CA, Athanasiou KA (2005). Effects of growth factors on meniscal fibrochondrocytes. *Tissue Engineering*.

[103] Huey DJ, Athanasiou KA (2011). Maturational growth of self-assembled, functional menisci as a result of TGF-*β*1 and enzymatic chondroitinase-ABC stimulation. *Biomaterials*.

[104] Ozkaynak E, Schnegelsberg PNJ, Jin DF (1992). Osteogenic protein-2. A new member of the transforming growth factor-*β* superfamily expressed early in embryogenesis. *The Journal of Biological Chemistry*.

[105] Wozney JM, Rosen V (1998). Bone morphogenetic protein and bone morphogenetic protein gene family in bone formation and repair. *Clinical Orthopaedics and Related Research*.

[106] Fukui N, Zhu Y, Maloney WJ, Clohisy J, Sandell LJ (2003). Stimulation of BMP-2 expression by pro-inflammatory cytokines IL-1 and TNF-*α* in normal and osteoarthritic chondrocytes. *Journal of Bone and Joint Surgery—Series A*.

[107] Shintani N, Hunziker EB (2007). Chondrogenic differentiation of bovine synovium: bone morphogenetic proteins 2 and 7 and transforming growth factor *β*1 induce the formation of different types of cartilaginous tissue. *Arthritis and Rheumatism*.

[108] Im HJ, Pacione C, Chubinskaya S, van Wijnen AJ, Sun Y, Loeser RF (2003). Inhibitory effects of insulin-like growth factor-1 and osteogenic protein-1 on fibronectin fragment- and interleukin-1*β*-stimulated matrix metalloproteinase-13 expression in human chondrocytes. *The Journal of Biological Chemistry*.

[109] Buma P, Ramrattan NN, van Tienen TG, Veth RPH (2004). Tissue engineering of the meniscus. *Biomaterials*.

[110] Longo UG, Olivia F, Denaro V, Maffulli N (2008). Oxygen species and overuse tendinopathy in athletes. *Disability and Rehabilitation*.

[111] Longo UG, Franceschi F, Loppini M, Maffulli N, Denaro V (2008). Rating systems for evaluation of the elbow. *British Medical Bulletin*.

[112] Longo UG, Garau G, Denaro V, Maffulli N (2008). Surgical management of tendinopathy of biceps femoris tendon in athletes. *Disability and Rehabilitation*.

[113] Longo UG, Ronga M, Maffulli N (2009). Achilles tendinopathy. *Sports Medicine and Arthroscopy Review*.

[114] Longo UG, Ronga M, Maffulli N (2009). Acute ruptures of the achilles tendon. *Sports Medicine and Arthroscopy Review*.

[115] Longo UG, Franceschi F, Ruzzini L (2009). Characteristics at haematoxylin and eosin staining of ruptures of the long head of the biceps tendon. *British Journal of Sports Medicine*.

[116] Longo UG, Franceschi F, Ruzzini L, Rabitti C, Nicola M, Denaro V (2009). Foreign-body giant-cell reaction at the donor site after autologous osteochondral transplant for cartilaginous lesion. A case report. *Journal of Bone and Joint Surgery—Series A*.

[117] Longo UG, Franceschi F, Ruzzini L, Spiezia F, Maffulli N, Denaro V (2009). Higher fasting plasma glucose levels within the normoglycaemic range and rotator cuff tears. *British Journal of Sports Medicine*.

[118] Kashyap AS, Anand KP, Kashyap S (2009). Minimally invasive total knee arthroplasty. *The New England Journal of Medicine*.

[119] Longo UG, Rittweger J, Garau G (2009). No influence of age, gender, weight, height, and impact profile in achilles tendinopathy in masters track and field athletes. *American Journal of Sports Medicine*.

[120] Longo UG, Maffulli N, Denaro V (2009). Rivaroxaban versus enoxaparin after total knee arthroplasty. *The Lancet*.

[121] Longo UG, Denaro V (2009). Spinal augmentation: what have we learnt?. *The Lancet*.

[122] Longo UG, Papapietro N, Maffulli N, Denaro V (2009). Thoracoscopy for minimally invasive thoracic spine surgery. *Orthopedic Clinics of North America*.

[123] Pufe T, Petersen WJ, Miosge N (2004). Endostatin/collagen XVIII—an inhibitor of angiogenesis—is expressed in cartilage and fibrocartilage. *Matrix Biology*.

[124] Petersen W, Pufe T, Stärke C (2005). Locally applied angiogenic factors—a new therapeutic tool for meniscal repair. *Annals of Anatomy*.

[125] Grotendorst GR, Martin GR, Pencev D (1985). Stimulation of granulation tissue formation by platelet-derived growth factor in normal and diabetic rats. *The Journal of Clinical Investigation*.

[126] Kirchberg K, Lange TS, Klein EC (1995). Induction of *β*1 integrin synthesis by recombinant platelet-derived growth factor (PDGF-AB) correlates with an enhanced migratory response of human dermal fibroblasts to various extracellular matrix proteins. *Experimental Cell Research*.

[127] Spindler KP, Mayes CE, Miller RR, Imro AK, Davidson JM (1995). Regional mitogenic response of the meniscus to platelet-derived growth factor (PDGF-AB). *Journal of Orthopaedic Research*.

[128] Tumia NS, Johnstone AJ (2009). Platelet derived growth factor-AB enhances knee meniscal cell activity in vitro. *Knee*.

[129] McNulty AL, Estes BT, Wilusz RE, Weinberg JB, Guilak F (2010). Dynamic loading enhances integrative meniscal repair in the presence of interleukin-1. *Osteoarthritis and Cartilage*.

[130] McNulty AL, Moutos FT, Weinberg JB, Guilak F (2007). Enhanced integrative repair of the porcine meniscus in vitro by inhibition of interleukin-1 or tumor necrosis factor *α*. *Arthritis and Rheumatism*.

[131] Longo UG, Fazio V, Poeta ML (2010). Bilateral consecutive rupture of the quadriceps tendon in a man with BstUI polymorphism of the COL5A1 gene. *Knee Surgery, Sports Traumatology, Arthroscopy*.

[132] Longo UG, Forriol F, Maffulli N, Denaro V (2010). Evaluation of histological scoring systems for tissue-engineered, repaired and osteoarthritic cartilage. *Osteoarthritis and Cartilage*.

[133] Longo UG, Franceschetti E, Maffulli N, Denaro V (2010). Hip arthroscopy: state of the art. *British Medical Bulletin*.

[134] Longo UG, Loppini M, Denaro L, Maffulli N, Denaro V (2010). Rating scales for low back pain. *British Medical Bulletin*.

[135] Longo UG, Lamberti A, Maffulli N, Denaro V (2010). Tendon augmentation grafts: a systematic review. *British Medical Bulletin*.

[136] Longo UG, Franceschi F, Spiezia F, Forriol F, Maffulli N, Denaro V (2010). Triglycerides and total serum cholesterol in rotator cuff tears: do they matter?. *British Journal of Sports Medicine*.

[137] Longo UG, Denaro L, Campi S, Maffulli N, Denaro V (2010). Upper cervical spine injuries: indications and limits of the conservative management in Halo vest. A systematic review of efficacy and safety. *Injury*.

[138] Longo UG, Buchmann S, Franceschetti E, Maffulli N, Denaro V A systematic review of single-bundle versus double-bundle anterior cruciate ligament reconstruction.

[139] Longo UG, Forriol F, Campi S, Maffulli N, Denaro V (2011). Animal models for translational research on shoulder pathologies: from bench to bedside. *Sports Medicine and Arthroscopy Review*.

[140] Longo UG, Buchmann S, Berton A, Maffulli N, Denaro V (2011). Arthroscopic knots and strength sutures for rotator cuff repair. *Sports Medicine and Arthroscopy Review*.

[141] Longo UG, Berton A, Ahrens PM, Maffulli N, Denaro V (2011). Clinical tests for the diagnosis of rotator cuff disease. *Sports Medicine and Arthroscopy Review*.

[142] Longo UG, Banerjee S, Barber J (2011). Conservative management versus open reduction and internal fixation for mid-shaft clavicle fractures in adults—the Clavicle Trial: study protocol for a multicentre randomized controlled trial. *Trials*.

[143] Longo UG, Berton A, Khan WS, Maffulli N, Denaro V (2011). Histopathology of rotator cuff tears. *Sports Medicine and Arthroscopy Review*.

[144] Longo UG, Rittweger J, Garau G (2011). Patellar tendinopathy in master track and field athletes: influence of impact profile, weight, height, age and gender. *Knee Surgery, Sports Traumatology, Arthroscopy*.

[145] Longo UG, Vasta S, Maffulli N, Denaro V (2011). Scoring systems for the functional assessment of patients with rotator cuff pathology. *Sports Medicine and Arthroscopy Review*.

[146] Longo UG, Denaro L, Spiezia F, Forriol F, Maffulli N, Denaro V (2011). Symptomatic disc herniation and serum lipid levels. *European Spine Journal*.

[147] Longo UG, Franceschi F, Spiezia F, Marinozzi A, Maffulli N, Denaro V (2011). The low-profile Roman bridge technique for knotless double-row repair of the rotator cuff. *Archives of Orthopaedic and Trauma Surgery*.

[148] Longo UG, Lamberti A, Maffulli N, Denaro V (2011). Tissue engineered biological augmentation for tendon healing: a systematic review. *British Medical Bulletin*.

[149] Longo UG, Marinozzi A, Cazzato L, Rabitti C, Maffulli N, Denaro V (2011). Tuberculosis of the shoulder. *Journal of Shoulder and Elbow Surgery*.

[150] Longo UG, Huijsmans PE, Maffulli N, Denaro V, De Beer JF (2011). Video analysis of the mechanisms of shoulder dislocation in four elite rugby players. *Journal of Orthopaedic Science*.

[151] Delos D, Rodeo SA (2011). Enhancing meniscal repair through biology: platelet-rich plasma as an alternative strategy. *Instructional Course Lectures*.

[152] Longo UG, Franceschi F, Berton A, Maffulli N, Denaro V (2012). Arthroscopic transosseous rotator cuff repair. *Medicine and Sport Science*.

[153] Longo UG, Berton A, Papapietro N, Maffulli N, Denaro V (2012). Biomechanics of the rotator cuff: european perspective. *Medicine and Sport Science*.

[154] Longo UG, Franceschi F, Berton A, Maffulli N, Denaro V (2012). Conservative treatment and rotator cuff tear progression. *Medicine and Sport Science*.

[155] Longo UG, Berton A, Papapietro N, Maffulli N, Denaro V (2012). Epidemiology, genetics and biological factors of rotator cuff tears. *Medicine and Sport Science*.

[156] Longo UG, Berton A, Marinozzi A, Maffulli N, Denaro V (2012). Subscapularis tears. *Medicine and Sport Science*.

[157] Longo UG, Lamberti A, Rizzello G, Maffulli N, Denaro V (2012). Synthetic augmentation in massive rotator cuff tears. *Medicine and Sport Science*.

[158] Maffulli N, Ajis A, Longo UG, Denaro V (2007). Chronic rupture of tendo achillis. *Foot and Ankle Clinics*.

[159] Maffulli N, Longo UG (2008). Conservative management for tendinopathy: is there enough scientific evidence?. *Rheumatology*.

[160] Maffulli N, Walley G, Sayana M, Longo UG, Denaro V (2008). Eccentric calf muscle training in athletic patients with Achilles tendinopathy. *Disability and Rehabilitation*.

[161] Maffulli N, Longo UG (2008). How do eccentric exercises work in tendinopathy?. *Rheumatology*.

[162] Ishida K, Kuroda R, Miwa M (2007). The regenerative effects of platelet-rich plasma on meniscal cells in vitro and its in vivo application with biodegradable gelatin hydrogel. *Tissue Engineering*.

[163] Maffulli N, Longo UG, Gougoulias N, Denaro V (2008). Ipsilateral free semitendinosus tendon graft transfer for reconstruction of chronic tears of the Achilles tendon. *BMC Musculoskeletal Disorders*.

[164] Maffulli N, Longo UG, Testa V, Oliva F, Capasso G, Denaro V (2008). Italian translation of the VISA-A score for tendinopathy of the main body of the Achilles tendon. *Disability and Rehabilitation*.

[165] Maffulli N, Longo UG, Franceschi F, Rabitti C, Denaro V (2008). Movin and bonar scores assess the same characteristics of tendon histology. *Clinical Orthopaedics and Related Research*.

[166] Maffulli N, Testa V, Capasso G (2008). Surgery for chronic Achilles tendinopathy produces worse results in women. *Disability and Rehabilitation*.

[167] Maffulli N, Longo UG, Testa V, Oliva F, Capasso G, Denaro V (2008). VISA-P score for patellar tendinopathy in males: adaptation to Italian. *Disability and Rehabilitation*.

[168] Maffulli N, Longo UG, Denaro V (2009). Anterior cruciate ligament tear. *The New England Journal of Medicine*.

[169] Maffulli N, Longo UG, Oliva F, Denaro V, Coppola C (2009). Bosch osteotomy and scarf osteotomy for hallux valgus correction. *Orthopedic Clinics of North America*.

[170] Maffulli N, Longo UG, Denaro V (2009). Complications after surgery or nonoperative treatment for acute achilles tendon rpture. *Clinical Journal of Sport Medicine*.

[171] Maffulli N, Longo UG, Denaro V (2009). Letter to the editor: minimally invasive paratenon release for non-insertional Achilles tendinopathy. *Foot & Ankle International*.

[172] Maffulli N, Longo UG, Oliva F, Ronga M, Denaro V (2009). Minimally invasive surgery of the achilles tendon. *Orthopedic Clinics of North America*.

[173] Maffulli N, Longo UG, Hüfner T, Denaro V (2010). Surgical treatment for pain syndromes of the Achilles tendon. *Unfallchirurg*.

[174] Maffulli N, Franceschi F, Longo UG, Ruzzini L, Denaro V (2010). Clinical evidence for suture anchor repair of rotator cuff tears does add up: some just do not want to see it. *Arthroscopy*.

[175] Maffulli N, Longo UG, Loppini M, Denaro V (2010). Current treatment options for tendinopathy. *Expert Opinion on Pharmacotherapy*.

[176] Maffulli N, Longo UG, Ronga M, Khanna A, Denaro V (2010). Favorable outcome of percutaneous repair of achilles tendon ruptures in the elderly. *Clinical Orthopaedics and Related Research*.

[177] Maffulli N, Longo UG, Spiezia F, Denaro V (2010). Free hamstrings tendon transfer and interference screw fixation for less invasive reconstruction of chronic avulsions of the Achilles tendon. *Knee Surgery, Sports Traumatology, Arthroscopy*.

[178] Maffulli N, Spiezia F, Longo UG, Denaro V (2010). Less-invasive reconstruction of chronic achilles tendon ruptures using a peroneus brevis tendon transfer. *American Journal of Sports Medicine*.

[179] Ritchie JR, Miller MD, Bents RT, Smith DK (1998). Meniscel repair in the goat model: the use of healing adjuncts on central tears and the role of magnetic resonance arthrography in repair evaluation. *American Journal of Sports Medicine*.

[180] Maffulli N, Longo UG, Gougoulias N, Loppini M, Denaro V (2010). Long-term health outcomes of youth sports injuries. *British Journal of Sports Medicine*.

[181] Maffulli N, Longo UG, Denaro V (2010). Novel approaches for the management of tendinopathy. *Journal of Bone and Joint Surgery—Series A*.

[182] Maffulli N, Longo UG, Spiezia F, Denaro V (2010). Sports injuries in young athletes: long-term outcome and prevention strategies. *Physician and Sportsmedicine*.

[183] Maffulli N, Longo UG, Maffulli GD, Khanna A, Denaro V (2011). Achilles tendon ruptures in diabetic patients. *Archives of Orthopaedic and Trauma Surgery*.

[184] Maffulli N, Longo UG, Maffulli GD, Khanna A, Denaro V (2011). Achilles tendon ruptures in elite athletes. *Foot and Ankle International*.

[185] Maffulli N, Longo UG, Spiezia F, Denaro V (2010). Aetiology and prevention of injuries in elite young athletes. *Medicine and Sport Science*.

[186] Maffulli N, Longo UG, Berton A, Loppini M, Denaro V (2011). Biological factors in the pathogenesis of rotator cuff tears. *Sports Medicine and Arthroscopy Review*.

[187] Maffulli N, Longo UG, Marinozzi A, Denaro V (2011). Hallux valgus: effectiveness and safety of minimally invasive surgery. A systematic review. *British Medical Bulletin*.

[188] Maffulli N, Longo UG, Maffulli GD, Rabitti C, Khanna A, Denaro V (2011). Marked pathological changes proximal and distal to the site of rupture in acute Achilles tendon ruptures. *Knee Surgery, Sports Traumatology, Arthroscopy*.

[189] Maffulli N, Longo UG, Gougoulias N, Caine D, Denaro V (2011). Sport injuries: a review of outcomes. *British Medical Bulletin*.

[190] Malliaropoulos N, Ntessalen M, Papacostas E, Longo UG, Maffulli N (2009). Reinjury after acute Lateral ankle sprains in elite track and field athletes. *American Journal of Sports Medicine*.

[191] Marinozzi A, Longo UG, Cazzato L, Martinelli N, Maffulli N, Denaro V (2011). Bilateral tibial hallux sesamoid agenesis and fibular hallux sesamoid hypoplasia in a patient with bilateral hallux valgus. *Journal of the American Podiatric Medical Association*.

[192] Martinelli N, Longo UG, Marinozzi A, Franceschetti E, Costa V, Denaro V (2011). Cross-cultural adaptation and validation with reliability, validity, and responsiveness of the Italian version of the Oxford Hip Score in patients with hip osteoarthritis. *Quality of Life Research*.

[193] Martinez de Albornoz P, Khanna A, Longo UG, Forriol F, Maffulli N The evidence of low-intensity pulsed ultrasound for in vitro, animal and human fracture healing.

[194] Nicolò M, Paolo R, Francesco C, Andrea M, Longo UG, Vincenzo D (2010). Hemiarthroplasty in a patient affected by osteonecrosis of the first metatarsal head following chevron osteotomy: a case report. *Foot*.

[195] Oliva F, Longo UG, Maffulli N (2009). Minimally invasive hallux valgus correction. *Orthopedic Clinics of North America*.

[196] Oliva F, Ronga M, Longo UG, Testa V, Capasso G, Maffulli N (2009). The 3-in-1 procedure for recurrent dislocation of the patella in skeletally immature children and adolescents. *American Journal of Sports Medicine*.

[197] Rizzello G, Franceschi F, Longo UG (2009). Arthroscopic management of calcific tendinopathy of the shoulder: do we need to remove all the deposit?. *Bulletin of the NYU Hospital for Joint Diseases*.

[198] Rizzello G, Longo UG, Franceschi F (2009). Compression neuropathy of the motor fibers of the median nerve at wrist level. *Journal of the Chinese Medical Association*.

[199] Rizzello G, Longo UG, Maffulli N, Denaro V (2010). Arthroscopic removal of an intraarticular osteoid osteoma of the distal tibia. *Journal of Foot and Ankle Surgery*.

[200] Ronga M, Oliva F, Longo UG, Testa V, Capasso G, Maffulli N (2009). Isolated medial patellofemoral ligament reconstruction for recurrent patellar dislocation. *American Journal of Sports Medicine*.

[201] Ronga M, Shanmugam C, Longo UG, Oliva F, Maffulli N (2009). Minimally invasive osteosynthesis of distal tibial fractures using locking plates. *Orthopedic Clinics of North America*.

[202] Ronga M, Longo UG, Maffulli N (2010). Minimally invasive locked plating of distal tibia fractures is safe and effective. *Clinical Orthopaedics and Related Research*.

[203] Thermann H, Gavriilidis I, Longo UG, Maffulli N (2009). Total ankle arthroplasty and tibialis posterior tendon transfer for ankle osteoarthritis and drop foot deformity. *Foot and Ankle Surgery*.

